# Predicting cardiovascular events in hemodialysis patients based on the fusion of physicochemical indicators and tongue images: a prospective and multicenter study

**DOI:** 10.3389/fphys.2026.1782190

**Published:** 2026-03-04

**Authors:** Kun Zou, Fan Xiao, Shuang Cheng, Qingxiang Wang, Xiaohua He, Jin Wang, Lijuan Dong, Kun Bao, Wu Zhou, Daixin Zhao

**Affiliations:** 1 School of Medical Information Engineering, Guangzhou University of Chinese Medicine, Guangzhou, China; 2 Department of Nephrology, Zhongshan Hospital of Traditional Chinese Medicine Affiliated to Guangzhou University of Chinese Medicine, Zhongshan, China; 3 Department of Nursing, Zhongshan Hospital of Traditional Chinese Medicine Affiliated to Guangzhou University of Chinese Medicine, Zhongshan, China; 4 State Key Laboratory of Dampness Syndrome of Chinese Medicine, Department of Nephrology, The Second Affiliated Hospital of Guangzhou University of Chinese Medicine (Guangdong Provincial Hospital of Chinese Medicine), Guangzhou, China; 5 Department of Nephrology, Guangdong Provincial Hospital of Chinese Medicine, Guangzhou, China

**Keywords:** cardiovascular events, feature fusion, hemodialysis patients, machine learning, tongue images

## Abstract

**Background:**

Cardiovascular events (CVEs) are the leading cause of mortality in hemodialysis patients. Current prediction models rely on clinical and biochemical data, but non-invasive alternatives are needed. Inspired by the Traditional Chinese Medicine (TCM) principle that “the heart opens into the tongue,” this study investigated whether quantitative features from tongue images could enhance CVE prediction.

**Objective:**

To develop and validate a machine learning framework that integrates tongue image features with conventional clinical variables to predict CVEs in hemodialysis patients.

**Methods:**

In this prospective, multicenter study, 506 maintenance hemodialysis patients were recruited. We extracted 1,354 hand-crafted radiomic features and 8 deep-learning features from standardized tongue images. These were combined with 90 clinical variables. Using a dataset split into training (n=243), validation (n=105), and an independent external test set (n=158), we developed and compared four models (LR, LightGBM, AdaBoost, MLP) under three feature configurations: clinical-only, tongue-only, and a fused model.

**Results:**

The model using only tongue image features (AdaBoost) significantly outperformed the clinical-only model, achieving an AUC of 0.786 vs. 0.682 on the external test set. The fused model provided a marginal improvement (AUC=0.787). SHAP analysis indicated that both tongue texture features and clinical biomarkers like PT% were key predictors. Decision curve analysis confirmed the clinical utility of the tongue-based and fused models across a range of risk thresholds.

**Conclusion:**

Tongue image features are potent, non-invasive predictors of CVEs in hemodialysis patients, offering performance superior to conventional clinical variables. This AI-driven approach validates the TCM theory and presents a promising supplementary tool for enhancing risk stratification in nephrology care.

## 1 Introduction

Hemodialysis patients are confronted with a significantly heightened burden of cardiovascular disease, with mortality rates reaching up to 20-fold higher than those of the general population (Luo et al., 2025). The reported prevalence of cardiovascular events (CVEs) exhibits variation: one study indicated that 30.6% of hemodialysis patients experienced CVEs (Yoshida et al., 2025), while a retrospective analysis reported an incidence of 11.59% among end - stage renal disease patients undergoing hemodialysis (Fei et al., 2025). Considering that CVEs are a primary cause of death in this patient population, the development of accurate predictive models is of utmost importance for the early identification of high-risk individuals and timely interventions. This can potentially reduce severe complications such as myocardial infarction and heart failure, improve survival rates, and alleviate the healthcare burden (Gan et al., 2024).

Predictive models for hemodialysis patients frequently utilize clinical, demographic, and laboratory data. Ensemble methods (e.g., XGBoost, Random Forest) have demonstrated robust performance (AUCs ranging from 0.76 to 0.89) (Sheng et al., 2020; Li et al., 2023; Wang et al., 2024; Matsubara et al., 2017; Mei et al., 2020; Qin et al., 2024; Zhang et al., 2022). Nevertheless, these models rely on invasive or conventional data sources. Recent research has explored non - invasive alternatives, including medical imaging and facial photographs (Bär et al., 2024; Knorr et al., 2022). In contrast, integrating tongue image features with machine learning presents a non - invasive and convenient approach rooted in TCM. The TCM principle “the heart opens into the tongue” implies that the tongue’s morphology may reflect cardiovascular health (Duan et al., 2024).

Advances in artificial intelligence, especially deep learning, have facilitated the extraction of quantitative features from tongue images. Emerging evidence supports the potential of these features for predicting cardiac diseases (Duan et al., 2024; Yunhu et al., 2023). However, no study has yet incorporated tongue imaging into the prediction of cardiovascular events for hemodialysis patients, presumably due to practical challenges: (a) the limited availability of specialized tongue imaging equipment in routine clinical settings; (b) the necessity for TCM - guided interpretation, which requires an integrative framework bridging Eastern and Western medicine; and (c) the inherently interdisciplinary nature of the work, which demands collaboration across clinical medicine, TCM, and AI.

**FIGURE 1:**
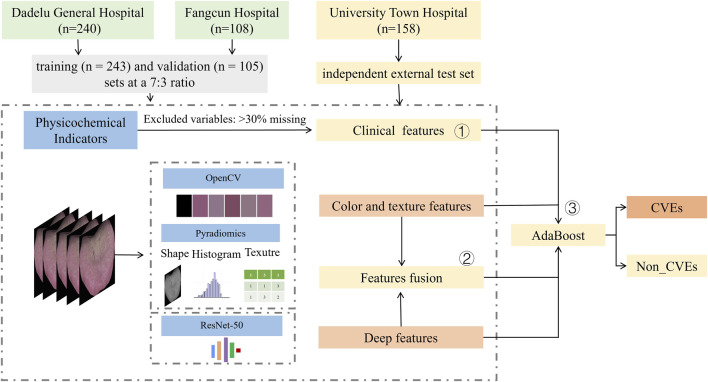
Flowchart of the study cohorts and design.

This study aims to employ machine learning to automatically extract key features from tongue images and develop a predictive model for cardiovascular events in hemodialysis patients. Furthermore, we propose integrating these tongue derived features with conventional clinical and laboratory parameters and validating the improved predictive performance in a prospective clinical study. This integrative approach aims to enhance the precision and personalization of cardiovascular risk stratification and intervention. The study flowchart is presented in Figure 1.

## 2 Materials and methods

### 2.1 Study population

This prospective, multicenter study recruited 506 maintenance hemodialysis patients from January 2024 to September 2025 across three branches of the Guangdong Provincial Hospital of Chinese Medicine, namely the University Town Hospital (n = 158), the Dadelu General Hospital (n = 240), and the Fangcun Hospital (n = 108). The study cohort consisted of 120 patients who had experienced cardiovascular events (CVE group) and 386 patients who had not (non-CVE group). The data from the Fangcun and Dadelu hospitals were randomly partitioned at a ratio of 7:3 into a training set (n = 243) and a validation set (n = 105). The data from the University Town Hospital (n = 158) were utilized as an independent external test set to assess the model’s generalizability.

Standardized tongue images were obtained using a specialized imaging device (Model DS01 B) during hemodialysis sessions, based on the traditional Chinese medicine tenet that “the heart opens to the tongue” (Huang and Yuan, 2020), which postulates that tongue morphology reflects cardiovascular health. Image acquisition was carried out under uniform lighting conditions to guarantee comparability among participants. In this research, tongue features are regarded as a non - invasive biomarker capable of detecting microcirculatory changes, such as those related to inflammation or fibrosis, which are associated with an increased cardiovascular risk in hemodialysis patients (Duan et al., 2024).

Simultaneously, 90 clinical and physicochemical variables were gathered, with laboratory values corresponding to the most recent test results available at the time of imaging. The study was approved by the hospital’s Ethics Committee (No. YE2024 022 01), and all participants provided written informed consent.

Inclusion criteria were as follows: (a) regular hemodialysis for ≥3 months; (b) age ≥18 years, conscious, and without major physical disabilities; (c) stable clinical condition with well - controlled comorbidities; and (d) ability to adhere to study procedures and provide reliable data. Exclusion criteria were: (a) active infection, aldosteronism, adrenal lesions, severe liver/brain/hematopoietic disorders, psychiatric conditions, or poor general health; and (b) history of kidney transplantation.

Two patients at the University Town Hospital were unable to cooperate with tongue image acquisition and were thus excluded from the study, and one patient at the Fangcun Hospital declined participation due to privacy concerns. No participants were lost to follow-up or withdrew from the study.

### 2.2 Definition and adjudication of cardiovascular events

In this study, CVEs were defined as the initial occurrence of any of the following during the follow-up period, in accordance with the 2020 American Heart Association guidelines and international consensus (Merchant et al., 2020): (a) Myocardial infarction (MI) (National Expert Committee on Rational Drug Use NHaFPC, 2019), diagnosed by ischemic manifestations, dynamic electrocardiogram (ECG) alterations (e.g., ST - segment elevation or new left bundle - branch block), and the rise or fall of high-sensitivity troponin T (hs-TnT) with at least one value exceeding the 99th percentile; (b) Cardiovascular (Cardiovascular Disease Group SoP and Chinese Medical Association, 2022) mortality, ascribed to acute coronary syndrome, heart failure, documented malignant arrhythmia, or sudden cardiac death, without any non-cardiac cause, based on medical records, death certificates, or autopsy findings; (c) Hospitalization for heart failure (Juan et al., 2024), meeting the European Society of Cardiology (ESC) criteria and necessitating an admission of ≥24 h; (d) Hospitalized unstable angina, diagnosed in the absence of elevated troponin but with ischemic symptoms and objective evidence of ischemia; and (e) Clinically significant arrhythmias (Zhao et al., 2020)—sustained ventricular tachycardia, hemodynamically compromising atrial fibrillation (e.g., systolic blood pressure <90 mmHg or requiring urgent cardioversion), or high-grade atrioventricular (AV) block (Mobitz II or third-degree) requiring intervention. Cerebrovascular events such as stroke were excluded from the primary endpoint. All suspected CVEs were independently adjudicated by two blinded cardiologists using clinical data, ECGs, serial hs - TnT measurements, and imaging modalities (e.g., echocardiography, angiography), in line with the current ESC/American College of Cardiology (ACC) criteria; discrepancies were resolved through consensus by a third senior cardiologist.

### 2.3 Data preprocessing

All preprocessing operations were carried out subsequent to dataset splitting to preclude data leakage. Preprocessing, encompassing missing value imputation, outlier removal, and normalization, utilized statistics exclusively derived from the training set. The parameters were uniformly applied to the validation and test sets. Among the 90 physicochemical variables, those with a missing value proportion exceeding 30% were excluded (Sterne et al., 2009) (The threshold selection is based on Reference (Sterne et al., 2009) to guarantee data integrity). The remaining missing values were imputed via Multiple Imputation by Chained Equations (MICE) in R (mice package v4.4.0), resulting in the generation of five imputed datasets. The final values were obtained by averaging across the imputations. Outliers deviating by more than ±3 standard deviations from the training set mean were removed.

Tongue images were visually examined by trained professionals. Images of poor quality (blurry, over/under - exposed, contaminated) were excluded. High - quality images were manually annotated using LabelMe (v5.2.1) to demarcate the tongue region. The JSON annotations were transformed into binary masks (using labelme_json_to_dataset) to segment the tongue and mask non - tongue areas (lips, skin, background), thereby minimizing visual noise during the training process.

Finally, all numerical features, including those derived from physicochemical data and tongue images, were standardized through Z - score normalization based on the training set means (μ) and standard deviations (σ), as shown in Equation 1. The same parameters were applied to the validation and test sets to ensure consistent evaluation and reproducibility.

$$z=\frac{x-\mu_{train}}{\sigma_{train}}$$ (1)

### 2.4 Algorithm selection

We conducted an evaluation of four algorithms: Logistic Regression (LR) with L2 regularization, LightGBM featuring moderate depth and strong L1/L2 regularization, AdaBoost employing shallow decision stumps, and a Multilayer Perceptron (MLP) equipped with a single hidden layer. Hyperparameters were adjusted in accordance with preliminary experiments to mitigate overfitting. All models were trained under three configurations: using only clinical variables, utilizing only tongue features, and incorporating fused features.

### 2.5 Implementation details

This research employed both hand - crafted and deep - learning techniques to extract multi - dimensional features from segmented tongue images. Hand - crafted features (n = 1,354) were calculated using OpenCV (v4.12.0) and PyRadiomics (v3.1.0), which included: (a) color descriptors (mean and standard deviation across RGB, CIELAB, HSV; 18 features); (b) texture metrics (GLCM, LBP, Gabor filters at 4 scales and 6 orientations); (c) shape descriptors (area, perimeter, eccentricity, and morphological measures from edge contours); and (d) Shannon entropy for textural complexity.

Regarding deep features, a ResNet - 50 model pre - trained on ImageNet was fine - tuned. Stochastic Gradient Descent (SGD) was utilized with a learning rate of 0.01, a momentum of 0.9, and a batch size of 32 over 30 epochs. Data augmentation (±15° rotation, horizontal flip, ±10% scaling) and cross - entropy loss were applied on an NVIDIA RTX 4090 GPU. The 2,048 - dimensional output from the penultimate layer was reduced to 8 dimensions through Principal Component Analysis (PCA), retaining components that accounted for ≥95% of the cumulative variance.

The final input consisted of 90 clinical/laboratory variables, 1,354 hand - crafted features, and 8 deep features (totaling 1,460 dimensions). To mitigate redundancy, LASSO regression with L1 regularization was employed. The optimal λ was selected via 10 - fold cross - validation using the one - standard - error rule. Only features with non - zero coefficients were retained. All features were Z - score normalized based on the training - set statistics prior to fusion. The entire pipeline was implemented in Python 3.9, leveraging opencv - python, PyRadiomics, scikit - learn, and PyTorch (v1.12.1).

### 2.6 Statistical analysis

All statistical analyses were carried out in Python 3.9, leveraging scipy (v1.13.1), statsmodels (v0.13.2), and scikit - learn (v1.0.2), with a two - sided significance level set at α = 0.05. Baseline characteristics were compared between patients with and without cardiovascular events (CVEs). Normality was evaluated through the Shapiro–Wilk test and Q–Q plots. For normally distributed continuous variables (characterized by homogeneous variance), they were summarized as the mean ± standard deviation (SD) and compared using the independent - samples t - test. In contrast, non - normal variables were reported as the median (inter - quartile range, IQR) and analyzed via the Mann–Whitney U test. Categorical variables were presented as counts (percentages) and compared using the chi - squared test; when the expected cell counts were <less than 5, Fisher’s exact test was employed.

To augment the robustness and validation of the model, sensitivity analyses were conducted. Specifically, 10 - fold cross - validation was implemented during LASSO hyperparameter tuning to alleviate overfitting. Uncertainty was quantified by calculating 95% confidence intervals for the area AUC using DeLong’s method, and further validation was achieved through 1,000 bootstrap resampling iterations.

The performance of the model on the independent test set was evaluated using multiple metrics, including the AUC, accuracy, sensitivity, specificity, positive and negative predictive values (PPV/NPV), and F1 - score. Calibration was assessed visually through calibration plots and statistically via the Hosmer–Lemeshow test (where P > 0.05 indicates an adequate fit). Clinical utility was determined through decision curve analysis (DCA), which measured the net benefit across risk thresholds ranging from 5% to 30%. A model was considered clinically useful if it outperformed both the “treat all” and “treat none” strategies.

## 3 Results

### 3.1 Training, validation, and test cohorts

This study encompassed 506 patients, with 348 patients from Fangcun and Dade Road General Hospitals constituting the model development cohort, and 158 patients from University Town Hospital serving as a completely independent external test cohort, which was not utilized at all during model development, hyper - parameter tuning, or data splitting.

Within the development cohort, stratified random splitting based on cardiovascular event status was carried out 10 times at a 7:3 ratio to maintain event prevalence and evaluate performance variability. For each iteration, models were trained on the training subset and evaluated on the validation subset, with the AUC serving as the primary evaluation metric. Although selecting the split with the highest performance may introduce a slight optimistic bias, this approach ensured consistency across analyses. The final conclusions were validated on the reserved external test set to minimize the risk of overfitting.

The split with the highest validation AUC was chosen as the fixed partition for all subsequent steps. It consisted of a training set of 243 patients (57 events, 23.5%) and a validation set of 105 patients (20 events, 19.0%). All subsequent procedures, including feature selection, hyper - parameter tuning, model comparison, and performance reporting, were strictly based on this fixed split to ensure reproducibility.

The external test cohort included 158 patients, 43 of whom experienced cardiovascular events (CVEs, (27.2%). The baseline characteristics were generally comparable to those of the development cohort (Table 1), indicating its suitability for unbiased external validation.

**TABLE 1:** Basic information of subjects.

Characteristic	Train cohort (n = 243)			Validation cohort (n = 105)			Test cohort (n = 158)		
	Non_CVEs	CVEs	P value	Non_CVEs	CVEs	P value	Non_CVEs	CVEs	P value
Age	58.194 ± 12.043	63.544 ± 12.265	0.014	57.035 ± 11.865	64.250 ± 12.234	0.014	55.409 ± 14.844	58.116 ± 13.672	0.358
AST	14.484 ± 8.879	14.544 ± 7.797	0.914	16.718 ± 12.709	12.750 ± 4.387	0.316	12.809 ± 9.356	11.558 ± 4.807	0.387
Ferritin	467.405 ± 531.074	286.761 ± 329.578	0.032	369.434 ± 459.053	207.100 ± 173.725	0.392	217.781 ± 255.778	163.598 ± 138.711	0.519
LDL_C	2.161 ± 0.876	1.989 ± 0.733	0.219	2.125 ± 0.727	2.592 ± 1.357	0.407	2.093 ± 0.871	1.850 ± 0.756	0.112
Cr	852.989 ± 290.288	744.860 ± 266.192	0.017	856.859 ± 276.065	727.150 ± 271.729	0.061	852.426 ± 300.809	885.000 ± 251.340	0.528
GLU	8.056 ± 3.908	8.936 ± 6.597	0.718	7.798 ± 3.181	8.309 ± 3.363	0.562	7.550 ± 3.679	8.927 ± 3.799	0.003
HCT	31.481 ± 6.078	31.279 ± 7.121	0.525	30.832 ± 7.340	29.110 ± 6.529	0.206	29.562 ± 6.560	31.070 ± 7.001	0.268
Ca	2.176 ± 0.199	2.167 ± 0.192	0.747	2.212 ± 0.229	2.146 ± 0.174	0.231	2.141 ± 0.213	2.133 ± 0.168	0.823
ALT	12.516 ± 11.048	12.386 ± 9.601	0.768	14.824 ± 12.435	9.850 ± 3.801	0.288	12.061 ± 13.287	10.023 ± 5.294	0.442
Hb	101.054 ± 18.398	100.386 ± 23.755	0.824	99.235 ± 22.763	92.400 ± 21.330	0.229	95.165 ± 21.446	99.581 ± 22.398	0.388
Fe	9.646 ± 5.192	9.724 ± 4.365	0.514	9.894 ± 5.285	8.094 ± 3.569	0.203	9.839 ± 3.896	10.668 ± 5.493	0.599
ALB	36.947 ± 4.962	37.782 ± 4.005	0.378	36.921 ± 4.304	36.995 ± 5.585	0.958	37.810 ± 4.680	39.109 ± 4.260	0.117
ALP	91.602 ± 69.891	90.421 ± 48.915	0.852	89.671 ± 53.784	83.850 ± 33.862	0.925	90.678 ± 62.346	90.233 ± 47.877	0.452
β2_MG	39.337 ± 12.052	42.704 ± 13.650	0.107	41.953 ± 13.930	44.891 ± 11.929	0.298	37.309 ± 14.674	42.481 ± 16.216	0.061
K	4.721 ± 0.845	4.698 ± 0.900	0.777	4.701 ± 0.752	4.631 ± 0.823	0.713	4.755 ± 0.743	4.749 ± 0.717	0.964
25_OH_D	53.432 ± 21.817	50.814 ± 21.554	0.448	53.311 ± 22.703	47.485 ± 20.579	0.219	77.220 ± 31.384	75.342 ± 29.967	0.936
AG	17.323 ± 5.408	17.333 ± 4.762	0.933	16.893 ± 4.767	17.330 ± 4.043	0.706	17.729 ± 3.989	18.953 ± 3.923	0.086
ALB/GLB	1.303 ± 0.288	1.319 ± 0.257	0.997	1.258 ± 0.279	1.260 ± 0.254	0.973	1.483 ± 0.351	1.542 ± 0.316	0.341
APTT	34.118 ± 8.771	35.614 ± 9.431	0.444	34.632 ± 8.712	34.690 ± 9.843	0.899	35.243 ± 9.950	37.635 ± 9.675	0.150
AST/ALT	1.312 ± 0.526	1.272 ± 0.450	0.751	1.300 ± 0.633	1.435 ± 0.574	0.247	1.268 ± 0.569	1.249 ± 0.459	0.682
BASO	0.037 ± 0.026	0.038 ± 0.022	0.436	0.042 ± 0.026	0.036 ± 0.023	0.391	0.036 ± 0.024	0.044 ± 0.037	0.358
BASO%	0.610 ± 0.387	0.665 ± 0.375	0.166	0.689 ± 0.494	0.600 ± 0.303	0.935	0.622 ± 0.512	0.672 ± 0.416	0.331
BNP	906.290 ± 1414.307	1672.230 ± 1866.926	0.005	1465.049 ± 1746.589	1569.825 ± 1787.118	0.771	1085.488 ± 1650.581	1514.847 ± 1696.087	0.018
CK	118.323 ± 137.508	132.544 ± 160.949	0.240	166.035 ± 219.761	149.100 ± 186.763	0.380	171.539 ± 207.477	185.395 ± 274.689	0.874
CK_MB	16.190 ± 7.683	16.561 ± 7.292	0.457	16.689 ± 7.634	17.790 ± 7.774	0.485	20.501 ± 10.177	17.914 ± 7.348	0.324
CL	99.366 ± 3.907	99.937 ± 4.670	0.358	98.594 ± 3.850	100.075 ± 2.998	0.111	100.403 ± 3.444	99.356 ± 3.289	0.087
CRP	7.132 ± 9.674	7.410 ± 9.655	0.814	7.115 ± 12.885	7.131 ± 7.077	0.110	6.587 ± 9.942	7.245 ± 9.759	0.296
eGFR	5.527 ± 3.020	6.540 ± 3.187	0.006	6.580 ± 10.128	6.615 ± 3.982	0.585	6.068 ± 5.122	5.146 ± 2.095	0.148
EOSIN	0.349 ± 0.448	0.330 ± 0.260	0.699	0.354 ± 0.295	0.231 ± 0.196	0.018	0.277 ± 0.220	0.384 ± 0.384	0.168
EOSIN%	5.361 ± 5.036	5.532 ± 3.990	0.552	5.659 ± 4.019	3.835 ± 2.659	0.044	4.795 ± 3.795	5.584 ± 4.150	0.230
FDP	2.806 ± 1.891	2.574 ± 1.201	0.688	3.032 ± 2.401	2.364 ± 0.943	0.241	6.748 ± 5.028	7.714 ± 5.471	0.274
FIB	3.722 ± 1.414	3.997 ± 1.515	0.360	3.972 ± 1.407	3.681 ± 1.538	0.238	4.105 ± 1.523	4.199 ± 1.462	0.863
GGT	30.199 ± 31.675	32.140 ± 24.497	0.277	33.106 ± 39.769	25.300 ± 14.769	0.845	22.026 ± 14.271	30.000 ± 40.900	0.385
GLB	29.194 ± 4.698	29.521 ± 4.341	0.640	29.924 ± 5.048	29.760 ± 4.201	0.993	26.462 ± 5.028	26.130 ± 4.754	0.882
HbA1c	5.909 ± 1.967	5.539 ± 1.355	0.870	6.172 ± 2.107	6.895 ± 2.322	0.027	6.143 ± 2.357	6.974 ± 2.803	0.213
HDL_C	1.086 ± 0.394	1.111 ± 0.351	0.573	1.116 ± 0.416	1.135 ± 0.407	0.613	0.946 ± 0.274	0.969 ± 0.315	0.983
hsCRP	10.017 ± 27.919	9.350 ± 17.464	0.646	8.470 ± 14.549	10.970 ± 23.325	0.568	7.125 ± 11.184	5.859 ± 5.413	0.676
INR	1.119 ± 0.237	1.101 ± 0.196	0.803	1.100 ± 0.220	1.082 ± 0.215	0.780	1.155 ± 0.263	1.140 ± 0.220	0.956
LDH	208.398 ± 62.560	211.526 ± 55.247	0.567	211.035 ± 66.096	182.150 ± 66.148	0.041	266.017 ± 92.647	241.791 ± 90.416	0.083
LYM	1.124 ± 0.412	1.136 ± 0.403	0.969	1.145 ± 0.393	1.080 ± 0.378	0.549	1.143 ± 0.448	1.177 ± 0.388	0.517
LYM%	18.728 ± 7.208	19.404 ± 6.900	0.595	18.859 ± 6.862	18.265 ± 6.549	0.654	19.563 ± 7.633	19.474 ± 5.612	0.774
MCH	29.061 ± 3.383	29.539 ± 2.814	0.605	28.804 ± 3.370	28.510 ± 3.203	0.967	28.773 ± 3.332	28.684 ± 3.129	0.418
MCHC	319.075 ± 10.556	320.544 ± 11.370	0.368	318.282 ± 12.742	317.050 ± 8.507	0.519	321.661 ± 12.801	320.814 ± 11.736	0.706
MCV	90.913 ± 9.429	92.123 ± 7.975	0.764	90.176 ± 8.943	89.850 ± 9.031	0.599	89.326 ± 8.697	89.330 ± 8.507	0.636
Mg	0.940 ± 0.161	0.932 ± 0.181	0.583	0.937 ± 0.184	0.917 ± 0.160	0.645	1.003 ± 0.134	1.006 ± 0.154	0.914
MONO	0.462 ± 0.219	0.435 ± 0.172	0.532	0.458 ± 0.168	0.487 ± 0.212	0.519	0.461 ± 0.222	0.443 ± 0.203	0.641
MONO%	7.362 ± 2.437	7.177 ± 2.143	0.542	7.274 ± 1.905	7.795 ± 2.390	0.582	7.630 ± 2.810	7.007 ± 2.010	0.312
MPV	9.184 ± 1.071	9.289 ± 0.968	0.506	9.265 ± 1.092	9.355 ± 1.342	0.751	9.494 ± 1.034	9.537 ± 1.121	0.819
Na	138.041 ± 3.047	138.000 ± 3.224	0.612	137.424 ± 3.503	137.900 ± 2.150	0.711	138.878 ± 2.596	138.163 ± 2.581	0.145
NEUT	4.356 ± 1.900	4.160 ± 1.485	0.806	4.427 ± 1.750	4.422 ± 1.784	0.948	4.321 ± 2.141	4.209 ± 1.537	0.930
NEUT%	67.849 ± 8.865	67.239 ± 9.582	0.942	67.373 ± 8.965	69.475 ± 6.976	0.329	67.398 ± 10.117	67.267 ± 7.149	0.493
nonHDL_C	2.694 ± 0.995	2.439 ± 0.800	0.125	2.621 ± 0.863	3.219 ± 1.517	0.222	2.744 ± 1.065	2.531 ± 0.998	0.206
P	1.875 ± 0.568	1.807 ± 0.628	0.251	1.884 ± 0.617	1.862 ± 0.781	0.546	1.761 ± 0.640	1.773 ± 0.632	0.910
PA	306.086 ± 83.597	290.632 ± 79.005	0.248	293.118 ± 80.854	307.600 ± 70.982	0.463	315.765 ± 81.712	322.233 ± 56.505	0.542
PCT	0.189 ± 0.059	0.183 ± 0.057	0.608	0.186 ± 0.066	0.208 ± 0.053	0.106	0.200 ± 0.083	0.204 ± 0.062	0.402
PDW	14.616 ± 2.410	14.232 ± 2.712	0.869	14.636 ± 2.280	14.705 ± 1.983	0.457	13.763 ± 2.729	14.812 ± 2.441	0.023
PLT	205.263 ± 69.753	198.053 ± 63.589	0.519	200.376 ± 70.181	227.400 ± 55.969	0.112	209.600 ± 80.178	220.465 ± 79.097	0.470
PT	13.949 ± 2.338	14.126 ± 2.118	0.620	14.205 ± 2.671	13.000 ± 2.362	0.061	13.677 ± 3.290	13.123 ± 3.372	0.186
PTH	443.239 ± 455.022	385.677 ± 343.393	0.959	391.642 ± 388.148	355.690 ± 316.270	0.880	434.369 ± 448.658	420.486 ± 323.302	0.361
PT%	80.238 ± 37.954	53.084 ± 34.677	<0.001	78.392 ± 38.240	60.935 ± 32.409	0.031	76.763 ± 37.924	58.263 ± 37.976	0.006
RBC	3.502 ± 0.707	3.402 ± 0.739	0.459	3.433 ± 0.713	3.259 ± 0.717	0.327	3.325 ± 0.736	3.488 ± 0.776	0.223
RDW	14.832 ± 1.889	14.495 ± 1.381	0.667	15.101 ± 2.110	15.430 ± 2.530	0.579	14.792 ± 1.918	14.779 ± 1.646	0.722
RET	64.372 ± 37.288	58.295 ± 35.615	0.100	61.091 ± 31.782	62.550 ± 37.462	0.659	25.163 ± 15.640	32.567 ± 27.520	0.253
RET%	1.797 ± 0.946	1.804 ± 1.124	0.527	1.826 ± 1.079	2.031 ± 1.537	0.899	0.794 ± 0.577	1.010 ± 0.817	0.086
TBA	6.216 ± 9.557	7.033 ± 9.757	0.237	7.142 ± 9.535	4.440 ± 3.023	0.141	4.890 ± 5.130	7.440 ± 14.874	0.571
TBIL	5.985 ± 2.237	5.907 ± 2.618	0.605	5.758 ± 2.121	5.440 ± 1.380	0.915	14.149 ± 19.765	6.637 ± 3.554	0.083
TC	3.780 ± 1.059	3.550 ± 0.858	0.187	3.743 ± 0.877	4.354 ± 1.609	0.298	3.691 ± 1.138	3.500 ± 0.982	0.422
TCO2	20.697 ± 3.540	21.212 ± 2.952	0.229	21.239 ± 3.304	20.555 ± 2.269	0.383	20.462 ± 3.012	19.935 ± 2.529	0.309
TG	1.616 ± 1.120	1.412 ± 0.998	0.038	1.837 ± 1.570	1.630 ± 0.996	0.732	1.727 ± 0.997	1.883 ± 1.560	0.958
TIBC	43.618 ± 11.025	45.674 ± 9.082	0.122	42.846 ± 12.030	45.255 ± 7.757	0.159	44.778 ± 9.652	45.400 ± 10.364	0.839
TnT	0.097 ± 0.094	0.112 ± 0.095	0.082	0.124 ± 0.129	0.113 ± 0.083	0.794	0.112 ± 0.114	0.081 ± 0.066	0.509
TP	66.232 ± 6.050	67.304 ± 5.375	0.211	66.448 ± 6.025	66.755 ± 6.755	0.842	64.271 ± 5.766	65.240 ± 5.995	0.270
TP_Ab	0.113 ± 0.128	0.131 ± 0.179	0.594	2.254 ± 19.904	0.132 ± 0.205	0.686	97.521 ± 91.889	68.430 ± 89.707	0.030
TSAT	23.510 ± 14.084	22.354 ± 11.224	0.840	24.059 ± 12.283	18.490 ± 9.576	0.060	23.071 ± 10.440	24.967 ± 14.620	0.693
TSH	13.510 ± 25.784	7.605 ± 19.938	0.034	15.793 ± 29.264	15.341 ± 29.119	0.229	34.000 ± 38.110	33.671 ± 38.120	0.785
TT	17.572 ± 2.726	18.149 ± 2.763	0.151	17.391 ± 2.709	18.430 ± 2.600	0.111	18.626 ± 2.786	18.784 ± 2.676	0.877
UA	435.366 ± 104.657	418.368 ± 112.228	0.293	414.106 ± 116.774	392.450 ± 105.198	0.380	394.583 ± 123.220	401.605 ± 85.714	0.555
UIBC	31.797 ± 13.323	33.532 ± 11.678	0.309	30.569 ± 14.084	34.855 ± 9.787	0.057	32.685 ± 11.930	33.579 ± 13.015	0.911
Urea	23.449 ± 7.205	21.812 ± 7.226	0.135	23.849 ± 6.655	23.172 ± 7.183	0.688	21.231 ± 6.679	21.971 ± 6.686	0.537
VB12	1305.656 ± 402.160	1295.368 ± 420.960	0.985	1304.553 ± 393.484	1190.750 ± 507.015	0.352	812.443 ± 559.750	1042.140 ± 551.268	0.036
WBC	6.331 ± 2.259	6.099 ± 1.740	0.805	6.426 ± 2.002	6.259 ± 2.153	0.654	6.237 ± 2.451	6.257 ± 2.135	0.967
Kt/V	1.231 ± 0.303	1.283 ± 0.338	0.255	1.228 ± 0.331	1.343 ± 0.269	0.160	1.423 ± 0.361	1.483 ± 0.290	0.265
URR	57.756 ± 11.699	58.471 ± 11.805	0.883	57.499 ± 12.297	57.851 ± 9.547	0.964	59.420 ± 9.443	59.754 ± 9.805	0.792
Dialysis.vintage	67.129 ± 68.668	61.228 ± 60.389	0.981	78.047 ± 75.644	53.300 ± 45.215	0.308	69.626 ± 53.497	52.744 ± 33.491	0.077
Sex			0.097			0.061			0.970
Female	94 (50.538)	21 (36.842)		33 (38.824)	13 (65.000)		45 (39.130)	16 (37.209)	
Male	92 (49.462)	36 (63.158)		52 (61.176)	7 (35.000)		70 (60.870)	27 (62.791)	
DM			0.566			0.406			0.294
N	166 (89.247)	53 (92.982)		78 (91.765)	20 (100.000)		105 (91.304)	42 (97.674)	
Y	20 (10.753)	4 (7.018)		7 (8.235)	Null		10 (8.696)	1 (2.326)	
Hypertension			0.867			0.303			0.278
N	138 (74.194)	41 (71.930)		60 (70.588)	17 (85.000)		85 (73.913)	36 (83.721)	
Y	48 (25.806)	16 (28.070)		25 (29.412)	3 (15.000)		30 (26.087)	7 (16.279)	
Hypotension			0.581			0.583			0.235
N	138 (74.194)	45 (78.947)		67 (78.824)	14 (70.000)		84 (73.043)	36 (83.721)	
Y	48 (25.806)	12 (21.053)		18 (21.176)	6 (30.000)		31 (26.957)	7 (16.279)	
Renal anemia			0.837			0.477			0.166
N	129 (69.355)	41 (71.930)		54 (63.529)	15 (75.000)		79 (68.696)	35 (81.395)	
Y	57 (30.645)	16 (28.070)		31 (36.471)	5 (25.000)		36 (31.304)	8 (18.605)	
ROD			0.932			1.000			0.193
N	147 (79.032)	46 (80.702)		65 (76.471)	15 (75.000)		86 (74.783)	37 (86.047)	
Y	39 (20.968)	11 (19.298)		20 (23.529)	5 (25.000)		29 (25.217)	6 (13.953)	

AG, anion gap; ALB, albumin; ALB/GLB, Albumin/Globulin Ratio; ALP, alkaline phosphatase; ALT, alanine aminotransferase; APTT, activated partial thromboplastin time; AST, aspartate aminotransferase; AST/ALT, Aspartate Aminotransferase/Alanine Aminotransferase Ratio; BASO, basophils; β2-MG, Beta-2, Microglobulin; BNP, B-type Natriuretic Peptide; CK, creatine kinase; Cr, Creatinine; CRP, C-reactive Protein; eGFR, estimated glomerular filtration rate; EOSIN, eosinophils; FDP, fibrin degradation products; FIB, fibrinogen; GLB, globulin; GLU, glucose; Hb, Hemoglobin; GGT, Gamma - Glutamyl Transferase; HbA1c, Hemoglobin A1c; HCT, hematocrit; HDL-C, High-Density Lipoprotein Cholesterol; hsCRP, High-Sensitivity C-reactive Protein; INR, international normalized ratio; LDH, lactate dehydrogenase; LDL-C, Low-Density Lipoprotein Cholesterol; LYM, lymphocytes; MCH, mean corpuscular hemoglobin; MCHC, mean corpuscular hemoglobin concentration; MCV, mean corpuscular volume; MONO, monocytes; MPV, mean platelet volume; NEUT, neutrophils; PA, prealbumin; PCT, procalcitonin; PDW, platelet distribution width; PLT, platelet count; PT, prothrombin time; PTH, parathyroid hormone; RBC, red blood cell count; RDW, red cell distribution width; RET, reticulocytes; TBA, total bile acids; TBIL, total bilirubin; TC, total cholesterol; TCO_2_, total carbon dioxide; TG, triglycerides; TIBC, Total Iron - Binding Capacity; TnT, Troponin T; TP, total protein; TP, Ab, Total Protein Antibody; TSAT, transferrin saturation; TSH, thyroid stimulating hormone; TT, total testosterone; UA, uric acid; UIBC, Unsaturated Iron - Binding Capacity; VB12, Vitamin B12; WBC, white blood cell count.

### 3.2 Model performance comparison

To conduct a systematic evaluation of the predictive value of diverse data modalities, a comparison was made regarding the performance of four machine - learning models, namely LR, LightGBM, AdaBoost, and MLP, across three feature configurations. These configurations included clinical variables solely, tongue image features solely, and a fused set integrating both. The performance was evaluated on an independent external test cohort, and the results are presented in Table 2.

**TABLE 2:** Performance of machine learning models by input feature type.

Model	Accuracy	AUC	95% CI	Sensitivity	Specificity	Precision	Recall	F1	Task
LR_clinical	0.733	0.769	0.7003–0.8376	0.702	0.742	0.455	0.702	0.552	Train
LR_clinical	0.620	0.670	0.5966–0.7434	0.730	0.585	0.357	0.730	0.479	Test
LightGBM_clinical	0.687	0.747	0.6782–0.8161	0.702	0.683	0.404	0.702	0.513	Train
LightGBM_clinical	0.582	0.628	0.5539–0.7013	0.651	0.560	0.318	0.651	0.427	Test
AdaBoost_clinical	0.733	0.783	0.7203–0.8449	0.702	0.742	0.455	0.702	0.552	Train
AdaBoost_clinical	0.696	0.682	0.6088–0.7560	0.524	0.750	0.398	0.524	0.452	Test
MLP_clinical	0.687	0.747	0.6762–0.8176	0.754	0.667	0.410	0.754	0.531	Train
MLP_clinical	0.567	0.660	0.5866–0.7336	0.810	0.490	0.333	0.810	0.472	Test
LR_tongue	0.691	0.856	0.8048–0.9073	0.912	0.624	0.426	0.912	0.581	Train
LR_tongue	0.806	0.750	0.6690–0.8313	0.667	0.850	0.583	0.667	0.622	Test
LightGBM_tongue	0.712	0.836	0.7785–0.8942	0.825	0.677	0.439	0.825	0.573	Train
LightGBM_tongue	0.684	0.759	0.6867–0.8306	0.778	0.655	0.415	0.778	0.541	Test
AdaBoost_tongue	0.778	0.876	0.8281–0.9248	0.895	0.742	0.515	0.895	0.654	Train
AdaBoost_tongue	0.810	0.786	0.7170–0.8557	0.714	0.840	0.584	0.714	0.643	Test
MLP_tongue	0.741	0.839	0.7863–0.8923	0.842	0.710	0.471	0.842	0.604	Train
MLP_tongue	0.719	0.683	0.5984–0.7670	0.667	0.735	0.442	0.667	0.532	Test
LR_fused	0.811	0.918	0.8787–0.9566	0.895	0.785	0.560	0.895	0.689	Train
LR_fused	0.764	0.776	0.7057–0.8454	0.651	0.800	0.506	0.651	0.569	Test
LightGBM_fused	0.811	0.873	0.8125–0.9336	0.842	0.801	0.565	0.842	0.676	Train
LightGBM_fused	0.757	0.772	0.7038–0.8401	0.698	0.775	0.494	0.698	0.579	Test
AdaBoost_fused	0.864	0.900	0.8506–0.9496	0.825	0.876	0.671	0.825	0.740	Train
AdaBoost_fused	0.745	0.787	0.7216–0.8530	0.730	0.750	0.479	0.730	0.579	Test
MLP_fused	0.811	0.897	0.8447–0.9495	0.842	0.801	0.565	0.842	0.676	Train
MLP_fused	0.757	0.779	0.7073–0.8513	0.683	0.780	0.494	0.683	0.573	Test

Models relying on tongue image features significantly outperformed those utilizing only clinical variables. The optimal clinical - only model (AdaBoost) attained a moderate AUC of 0.682, with a balanced sensitivity of 52.4% and specificity of 75.0%. However, it exhibited limited overall accuracy (69.6%) and F1-score (0.452). In contrast, the tongue - only AdaBoost model achieved a substantially higher AUC of 0.786, accompanied by enhanced accuracy (81.0%), sensitivity (71.4%), specificity (84.0%), and F1 - score (0.643), which demonstrated the robust discriminative capacity of non - invasive tongue imaging features.

The fusion of clinical and tongue features led to further, albeit marginal, improvements. The fused AdaBoost model achieved the highest AUC in this study (0.787), indicating a slight incremental value from multimodal integration. Nevertheless, this was accompanied by a trade - off in accuracy (74.5%) and F1 - score (0.579), primarily attributable to an increase in false positives. Other fused models (LR, MLP) showed consistent improvements over clinical-only models and modest enhancements in overall discrimination, albeit with a slight trade-off in specificity compared to tongue-only counterparts. For instance, the specificity of LR decreased from 85.0% (tongue-only) to 80.0% (fused), while the AUC increased from 0.750 to 0.776.

Notably, while the training performance was high across all models (e.g., training AUCs >0.85 for tongue and fused models), the test performance declined, particularly for MLP, suggesting some degree of overfitting. This is likely due to the high feature dimensionality relative to the sample size. Despite this, DCA indicated a positive net benefit across clinically relevant risk thresholds (5%–30%) for all tongue - based and fused models, supporting their potential clinical applicability.

**FIGURE 2:**
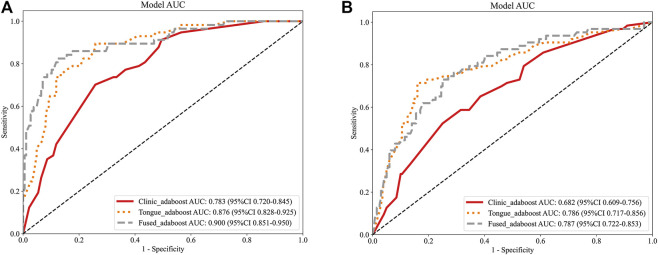
**(A)** ROC curves of the three models in the training cohort. **(B)** ROC curves of the three models in the test cohort.

In conclusion, tongue image features alone outperformed conventional clinical variables in predicting cardiovascular events in hemodialysis patients. Multimodal fusion provided a small yet consistent improvement in discrimination, with AdaBoost emerging as the most balanced and effective algorithm across all configurations. As depicted in the ROC curves (Figures 2A,B), the performance gap between the training and test sets suggests potential overfitting, which was alleviated through cross - validation.

### 3.3 Performance comparison

As presented in Table 2, the tongue image–based model (Tongue_all) attained a notably higher AUC value of 0.786 in comparison to the clinical model, which had an AUC of 0.682. This finding suggests that tongue features offer incremental predictive value. For example, when combined with tongue features, the AdaBoost model achieved a sensitivity of 71.4%, outperforming the model relying solely on clinical data, which had a sensitivity of 52.4%. This advantage is likely attributable to the tongue’s capacity to capture microvascular alterations that cannot be directly measured by conventional clinical variables.

**FIGURE 3:**
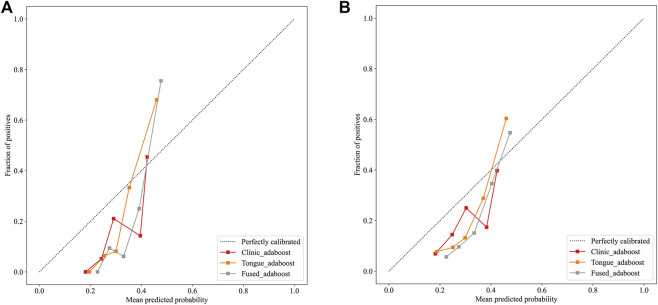
**(A)** Calibration plots of the three models in the training cohort. **(B)** Calibration plots of the three models in the test cohort.

**FIGURE 4:**
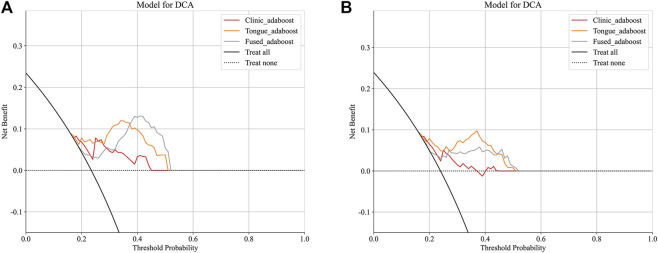
**(A)** DCA plots of the three models in the training cohort. **(B)** DCA plots of the three models in the test cohort.

**FIGURE 5:**
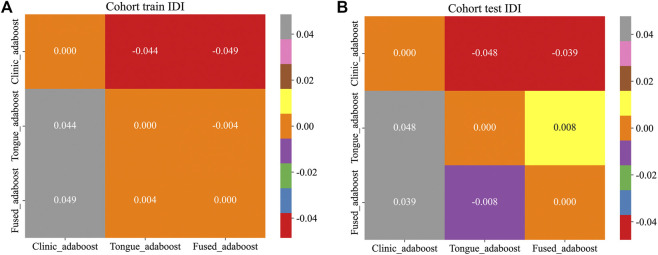
**(A)** IDI plots of the three models in the training cohort. **(B)** IDI plots of the three models in the test cohort.

The Tongue_all model, which combines deep learning and radiomic features, achieved the highest accuracy of 81.0% and an F1 - score of 0.643, with an AUC of 0.786. The Fused model exhibited a slight increase in AUC (0.787), yet demonstrated lower accuracy (74.5%) and specificity (75.0%), indicating a trade - off with a higher rate of false positives. The clinic - only model performed the least effectively, with an AUC of 0.682. DCA and Integrated Discrimination Improvement (IDI) analyses confirmed the superior clinical utility and incremental value of tongue - derived features (Table 3; Figures 2–5).

**TABLE 3:** Performance comparison of clinic-only, tongue-only, and fused models across training and test cohorts.

Model	Accuracy	AUC	95% CI	Sensitivity	Specificity	Precision	Recall	F1	Cohort
Clinic	0.733	0.783	0.7203–0.8449	0.702	0.742	0.455	0.702	0.552	Train
Tongue_DL	0.770	0.861	0.8105–0.9121	0.895	0.731	0.505	0.895	0.646	Train
Tongue_RAD	0.840	0.878	0.8297–0.9273	0.789	0.855	0.625	0.789	0.698	Train
Tongue_all	0.778	0.876	0.8281–0.9248	0.895	0.742	0.515	0.895	0.654	Train
Fused	0.864	0.900	0.8506–0.9496	0.825	0.876	0.671	0.825	0.740	Train
Clinic	0.696	0.682	0.6088–0.7560	0.524	0.750	0.398	0.524	0.452	Test
Tongue_DL	0.776	0.766	0.6930–0.8397	0.746	0.785	0.522	0.746	0.614	Test
Tongue_RAD	0.791	0.781	0.7120–0.8506	0.698	0.820	0.550	0.698	0.615	Test
Tongue_all	0.810	0.786	0.7170–0.8557	0.714	0.840	0.584	0.714	0.643	Test
Fused	0.745	0.787	0.7216–0.8530	0.730	0.750	0.479	0.730	0.579	Test

### 3.4 Important feature analysis and clinical interpretation

SHAP analysis demonstrated that both tongue texture features (such as wavelet - derived metrics) and clinical biomarkers (especially PT%) were the key determinants of prediction. Elevated values of specific texture features were correlated with an augmented risk, possibly mirroring underlying inflammation or microvascular alterations. In contrast, a higher PT% (signifying superior coagulation function) exerted a protective effect. The substantial influence of texture metrics corroborates the TCM notion that tongue morphology serves as an indicator of systemic health. This interpretability narrows the disparity between model output and clinical comprehension.

## 4 Discussion

In this prospective, multicenter study, we developed a machine learning framework for predicting cardiovascular events in maintenance hemodialysis patients by integrating quantitative tongue image features with conventional physicochemical indicators. Based on the TCM principle that “the heart opens into the tongue” (Huang and Yuan, 2020), this research combines the ancient diagnostic wisdom with modern data science, transforming a clinical observation into a testable, predictive model. This study serves as a “translational bridge”—not by directly resolving theoretical discrepancies, but by using artificial intelligence (AI) to quantify TCM signs. To our knowledge, this is the first prospective study to demonstrate that objective, quantifiable features of the tongue—assessed through digital imaging—can independently predict adjudicated cardiovascular outcomes in a high - risk renal population with clinically significant performance.

The superior performance of models incorporating tongue features compared to those based solely on clinical variables highlights a crucial gap in current risk stratification: conventional biomarkers, although informative, often fail to capture the complex, non - traditional pathophysiology prevalent in end - stage renal disease. The limited discriminatory ability of clinical - only models is consistent with the well - documented limitations of traditional risk scores such as Framingham (Dufouil et al., 2017) and ASCVD (Grundy et al., 2019) in dialysis populations, where inflammation, autonomic dysfunction, and microvascular disease play disproportionately large roles. The retention of established predictors like age, Ferritin, and creatinine by LASSO selection supports the biological plausibility of the baseline model (Figure 9), yet also emphasizes the necessity for complementary data sources to improve precision.

**FIGURE 6:**
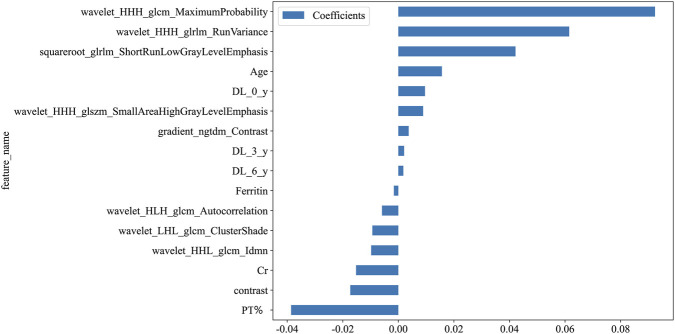
Feature coefficients from the fitted logistic regression model, ranked by magnitude.

Our approach utilizes the tongue not merely as an anatomical structure, but as a theory - driven, organ - specific biomarker reflecting systemic cardiovascular health (Wang et al., 2018). In TCM, the tongue is regarded as a window to internal organ function, particularly that of the heart and circulation. The strong predictive power of tongue - derived features—especially those extracted by deep learning—suggests that subtle morphological patterns, potentially indicative of microcirculatory impairment or chronic inflammation, are encoded in its visual appearance. While prior studies have associated facial or oral phenotypes with vascular aging (Mizukoshi et al., 2024; Mekić et al., 2021), this research uniquely validates the tongue as a non - invasive, theory - based indicator against hard clinical endpoints in a vulnerable population.

We further demonstrated the value of multimodal integration by combining two complementary sources of tongue information: radiomic features, which capture engineered descriptors of texture and heterogeneity, and deep learning features, which automatically extract hierarchical representations from raw images. The superior performance of the combined “Tongue_all” model indicates that these modalities are not redundant but synergistic. Notably, the strong sensitivity and competitive performance of deep learning features challenge the assumption that handcrafted radiomics are inherently more interpretable or effective, suggesting that data - driven models can detect subtle, clinically relevant patterns beyond the scope of predefined metrics.

The integration of tongue imaging with clinical data led to consistent, incremental improvements in predictive accuracy and clinical net benefit, as verified by decision curve analysis and measures of risk reclassification (IDI,NRI). This synergy likely results from the orthogonality of the data sources: laboratory values reflect systemic biochemical states, while tongue images capture localized, morphological manifestations that may represent early - stage microvascular or inflammatory changes not yet detectable in blood tests. This multimodal strategy enhances risk stratification by providing a more comprehensive view of the patient’s physiological state.

**FIGURE 7:**
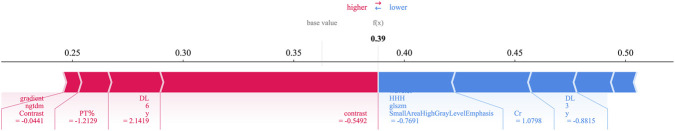
SHAP force plot for sample #150.

**FIGURE 8:**
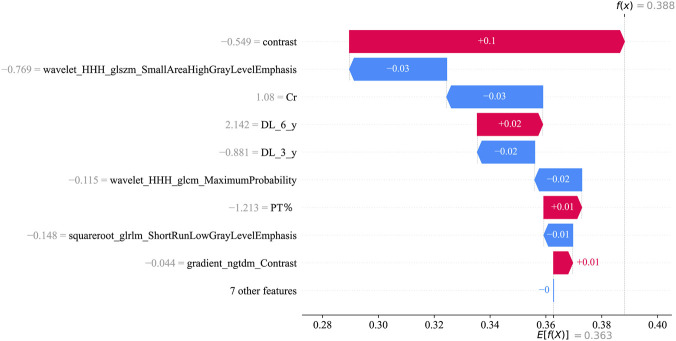
SHAP waterfall plot for sample #150.

**FIGURE 9:**
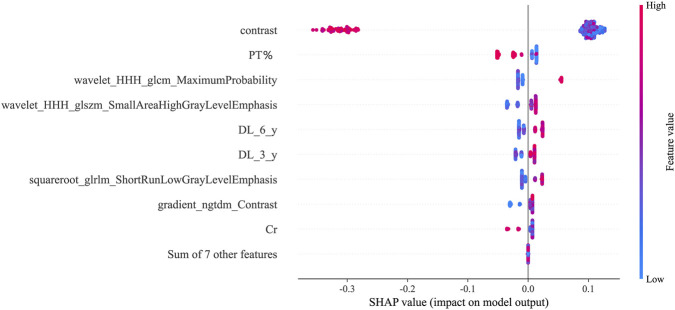
SHAP summary plot.

Decision curve analysis (Figures 4A,B) further indicated that the tongue - based model generates net clinical benefits across risk thresholds ranging from 5% to 30%, which validates its efficacy in early risk stratification. Consequently, we propose integrating standardized tongue imaging into routine dialysis evaluations, in conjunction with laboratory markers such as BNP, to facilitate personalized and proactive interventions. SHAP analysis (Figures 7–9) revealed that tongue texture features, such as wavelet - based metrics, are correlated with the risk of cardiovascular events, potentially reflecting the microcirculatory dysfunction and chronic inflammation frequently observed in hemodialysis patients (Sheng et al., 2020). For example, higher texture values are associated with irregularities on the tongue surface, which have been associated with myocardial fibrosis. This provides quantitative evidence for the TCM theory that “the heart opens to the tongue” and showcases the potential of tongue imaging as a quantifiable biomarker.

Several limitations deserve consideration. Although the sample size (n = 506) is reasonable for a multicenter study, the absence of an *a priori* power analysis and the high dimensionality of tongue imaging features—evidenced by the notable decline in MLP performance on the test set (AUC: 0.683)—raise concerns about overfitting and limit generalizability, highlighting the need for validation in larger, more diverse cohorts. Moreover, the analysis did not stratify patients by TCM syndrome patterns (e.g., Qi deficiency with blood stasis), potentially obscuring important subgroup differences. This study relied solely on single - time - point tongue images due to device availability, whereas longitudinal imaging capturing dynamic changes across the dialysis cycle or in response to treatment could enable real - time monitoring and early intervention. Importantly, tongue imaging is not intended to replace established diagnostics such as cardiac imaging but to complement them; future research should explore multimodal integration—combining tongue features with laboratory data, cardiac imaging, and other TCM diagnostic modalities (e.g., pulse diagnosis, complexion assessment, and symptom patterns)—to further enhance predictive accuracy and clinical utility.

## 5 Conclusion

In summary, this study introduces a novel artificial intelligence (AI)-driven multimodal framework that integrates quantitative tongue image features with clinical data to predict cardiovascular events in hemodialysis patients. This approach exhibits a significant advantage over unimodal models and demonstrates high clinical utility for risk stratification. Based on the TCM principle that “the heart is externally manifested on the tongue,” this research validates tongue morphology as a non-invasive biomarker that offers supplementary pathophysiological insights beyond conventional laboratory measurements. By combining both radiomic and deep learning-derived tongue features, this study reveals superior performance compared to models utilizing only one type of tongue feature. Future research will concentrate on enhancing model robustness and promoting clinical translation through multicenter validation and prospective studies, thereby laying the foundation for AI-powered, non-invasive precision medicine in nephrology.

## Data Availability

The raw data supporting the conclusions of this article will be made available by the authors, without undue reservation.
